# Potential of 3D Skin Models and N/TERT‐2G Cell Line in Genetic Research on Autosomal Recessive Nonsyndromic Epidermal Differentiation Disorders

**DOI:** 10.1111/exd.70298

**Published:** 2026-06-15

**Authors:** Hao‐Hsiang Hsu‐Rehder, Cristina Glocker, Christine Aldrian, Ezgi Dikici, Gesa Sophie Wilde, Mariana Stavila, Nathalie Jonca, Branislav Kollár, Steffen Ulrich Eisenhardt, Judith Fischer

**Affiliations:** ^1^ Institute of Human Genetics, Medical Center—University of Freiburg, Faculty of Medicine University of Freiburg Freiburg Germany; ^2^ Competence Center for Cornification Disorders, Freiburg Center for Rare Diseases University of Freiburg Freiburg Germany; ^3^ Toulouse Institute for Infectious and Inflammatory Diseases Toulouse University, CNRS, Inserm Toulouse France; ^4^ Department of Cell Biology and Cytology, Federative Institute of Biology, Purpan Hospital University Hospital Toulouse France; ^5^ Department of Plastic and Hand Surgery, Faculty of Medicine Medical Center – University of Freiburg Freiburg Germany

**Keywords:** 3D skin model, autosomal recessive congenital ichthyosis, autosomal recessive nonsyndromic epidermal differentiation disorders, epidermis skin equivalent, N/TERT‐2G, non‐syndromic ichthyosis

## Abstract

Autosomal recessive nonsyndromic epidermal differentiation disorders (AR‐nEDDs), also known as autosomal recessive congenital ichthyosis (ARCI), are rare genetic skin diseases that lack curative treatments and can only be managed symptomatically. Their study is hampered by the limited availability of biological samples and donor tissues. Artificial skin models offer a valuable alternative for experimental research. Here, we generated epidermal skin equivalents (ESE) using primary keratinocytes from patients with AR‐nEDDs carrying pathogenic variants in *ALOX12B*, *CYP4F22*, and *CERS3*, as well as immortalized N/TERT‐2G cells. Whereas primary cells undergo senescence, N/TERT‐2G cells offer a promising alternative to overcome this limitation. Histological staining and qPCR were applied to assess key epidermal proteins and AR‐nEDD‐related gene expression in patient‐ and control‐derived models. Patient‐derived ESE reproduced the major histopathological features and protein expression patterns characteristic of AR‐nEDDs. In contrast, N/TERT‐2G‐based models showed earlier expression of 12R‐LOX, CYP4F22, and CERS3 proteins in epidermal layers compared to healthy donor equivalents. These findings suggest that N/TERT‐2G cells represent a reproducible platform for future gene‐editing approaches of modelling epidermal differentiation disorders. However, as they do not inherently carry pathogenic variants, they are particularly suitable for gene‐editing approaches such as CRISPR/Cas9‐mediated disease modelling. Conversely, patient‐derived keratinocyte models remain indispensable for validating disease mechanisms and evaluating therapeutic strategies.

## 1 Introduction

Autosomal recessive nonsyndromic epidermal differentiation disorders (AR‐nEDDs) [1, 2], also referred to as autosomal recessive congenital ichthyosis (ARCI), are a group of rare hereditary disorders that predominantly affect the skin and are characterized by abnormal keratinization, epidermal hyperproliferation, dermal inflammation, and scaling [3]. AR‐nEDDs are typically generalized and affect the entire skin surface. The estimated incidence of these disorders ranges from 1:100 000 to 1.7:100 000 in Europe [3, 4, 5]. To date, 12 genes—*ABCA12, ALOX12B, ALOXE3, CASP14, CERS3, CYP4F22, NIPAL4, PNPLA1, SDR9C7, SULT2B1, LIPN*, and *TGM1*—have been established with AR‐nEDDs [1, 3, 5, 6, 7].

Currently, there is no cure for these disorders, which emphasizes the critical need for research in this field. However, such research requires skin biopsies from affected individuals, which presents a significant challenge due to both the limited size of samples and their restricted availability. One promising alternative to overcome these limitations is the use of artificial skin models. Over the years, 3D skin models have gained increasing importance—not only due to scientific advances but also in response to the 3Rs principle (Replacement, Reduction, and Refinement of animal use) and European regulations that restrict animal testing in the cosmetics industry [8].

In the context of skin disease, 3D skin models offer a valuable platform for developing and testing therapeutic strategies [9, 10, 11]. Their advantages include commercial availability and the possibility of incorporating patient‐derived cells [12]. Skin models vary in complexity; some protocols reconstruct full‐thickness skin equivalents comprising both epidermis and dermis, while others focus solely on the epidermis. Previous studies have successfully employed 3D skin models to investigate AR‐nEDDs, such as the work by Heinz et al. on *SULT2B1* [13] and Youssefian et al. on *SDR9C7* [14].

Another major challenge in studying genetic skin disorders is the limited lifespan of primary keratinocytes due to cellular senescence. The immortalized keratinocyte cell line N/TERT‐2G offers a promising alternative to address this limitation. Compared to the more widely used HaCaT cell line, N/TERT‐2G cells exhibit fewer chromosomal abnormalities [15], and have been successfully employed in generating 3D skin models and studying human skin diseases [16, 17]. Immortalized cells are indispensable for future applications such as gene‐edited disease models created using CRISPR/Cas9 technologies. In this context, we also aimed to explore the suitability of N/TERT‐2G cells for such future applications. The N/TERT‐2G cell line could serve as a control model for research on AR‐nEDDs and holds potential for gene‐editing approaches.

To evaluate 3D skin models in the context of AR‐nEDDs, we conducted experiments using a range of keratinocytes carrying pathogenic variants from our cohort and the N/TERT‐2G cell line. We generated epidermal equivalents and assessed their morphology and protein expression profiles. In addition, we performed 2D differentiation assays to analyse the mRNA expression of the late differentiation markers filaggrin and involucrin, as well as the AR‐nEDDs‐associated proteins 12R‐LOX, CYP4F22, and CERS3.

The three investigated genes—*ALOX12B, CYP4F22, CERS3*—encode enzymes involved in the ceramide metabolism of the epidermis [18, 19, 20, 21]. Disruptions in these processes can impact the expression and function of proteins such as filaggrin and involucrin, as well as the production of glucosylceramides, which are all crucial for maintaining the skin barrier. Therefore, we specifically focused our analyses on filaggrin, involucrin, and glucosylceramides.

## 2 Materials and Methods

### 2.1 Ethics Statement

The collection of patient samples was approved by the Institutional Ethical Review Board of the University of Freiburg (reference number: 436/17). Written informed consent was obtained from all participants prior to sample collection. All procedures were performed in accordance with the principles of the Declaration of Helsinki.

### 2.2 Cell Isolation and Culture

Primary keratinocytes were isolated from skin biopsies obtained from voluntary donors. Healthy skin was obtained as discarded tissues from patients undergoing body contouring surgery procedures at the Department of Plastic and Hand Surgery. The biopsies were first washed in 70% EtOH, followed by incubation in Dulbecco's Modified Eagle Medium (DMEM; Gibco, Carlsbad, USA) supplemented with 1% penicillin/streptomycin (P/S; anprotec, Bruckberg, Germany) and then immersed in Dulbecco's phosphate‐buffered saline (DPBS; Gibco, Carlsbad, USA). Subsequently, the skin was cut into small pieces and incubated overnight at 4°C in 5 mg/mL Dispase (Gibco, Carlsbad, USA) in DMEM to facilitate the separation of the epidermis from the dermis. Epidermal pieces were incubated in trypsin (anprotec, Bruckberg, Germany) at 37°C for 15 min. The pieces were gently separated with tweezers and repeatedly resuspended to release the keratinocytes from the epidermis. Keratinocytes were transferred to culture dishes and maintained in EpiLife medium (Thermo Scientific, Massachusetts, USA) supplemented with 1% human keratinocyte growth supplement (HGKS; Thermo Scientific, Massachusetts, USA) and 0.25% P/S (PAN‐Biotech, Aidenbach, Germany). After 2–3 days E1 medium (EpiLife + HKGS + 1% P/S) was added.

All cells were cultured at 37°C in a humidified incubator with 5% CO_2_. The culture medium was changed three times per week. Once cells reached 80% confluence, they were harvested, cryopreserved in FBS supplemented with 10% dimethyl sulfoxide (DMSO; Carl Roth, Karlsruhe, Germany) and stored in liquid nitrogen.

### 2.3 Keratinocytes

For this study, we used primary keratinocytes from a healthy donor (female, 29 years old, femoral biopsy) as control, the N/TERT‐2G cell line and primary keratinocytes from three different patients with various AR‐nEDD‐related variants from our cohort. Patient 1 (P1‐ALOX12B) carries a compound heterozygous variant in the *ALOX12B* gene. Specifically, the mutation c.299C>T in exon 2 results in the missense substitution p.Phe99Leu, while the intronic variant c.1655‐7C>A is predicted to affect normal RNA splicing, potentially leading to exon skipping or intron retention and subsequent protein instability. Patient 2 (P2‐CYP4F22) harbours a homozygous nonsense mutation in the *CYP4F22* gene. The mutation is located in exon 9 and introduces a premature stop codon, which may lead to nonsense‐mediated mRNA decay or result in the expression of a truncated protein. Patient 3 (P3‐CERS3) carries a homozygous splice site mutation in the *CERS3* gene affecting intron 9 (see Table 1). This variant has previously been described, with no detectable full‐length or truncated CERS3 protein observed in mutant keratinocytes, suggesting a loss of protein expression, possibly due to nonsense‐mediated mRNA decay or instability of the aberrant transcript.

**TABLE 1:** Overview of keratinocyte samples derived from AR‐nEDD patients used in this study.

Patient abbreviation	Gene	Gender/Age/Location	Variant	Chromosome	References
P1‐ALOX12B	*ALOX12B (DNA: NC_000017.11)*	Male/1/abdomen	c.299C>T, c.1655‐7C>A	17p13.1	18
P2‐CYP4F22	*CYP4F22 (DNA: NC_000019.10)*	Female/22/buttock	c.976C>T, c.976C>T	19p13.12	19
P3‐CERS3	*CERS3 (DNA: NC_000015.10)*	Male/32/abdomen	c.609+1G>T, c.609+1G>T	15q26.3	20

### 2.4 Generation of Epidermis Skin Equivalent

To generate the epidermal skin equivalent (ESE) models, 24‐well cell culture inserts (Nunc 0.4 μm PC pore size, Thermo Scientific, Massachusetts, USA) were coated with 0.1 mg/mL collagen A (PAN‐Biotech, Aidenbach, Germany) diluted in ddH_2_O and incubated for at least 1 h at 37°C. Subsequently, 3.53 × 10^5^ cells in 500 μL Eg1 Medium (E1 supplemented with 10 ng/mL keratinocyte growth factor (KGF; PeptroTech, Hamburg, Germany) and 0.14 mM CaCl_2_ (Sigma‐Aldrich, Missouri, USA) referred to as Eg1) were applied to each cell culture insert. The cells were cultured submerged at 37°C with 5% CO_2_ for 2 days. After 2 days, the cell culture inserts were transferred to air‐liquid interface conditions, and the basal medium was replaced with Eg2 medium (E1 supplemented with 10 ng/mL KGF and 1.7 mM CaCl_2_ referred to as Eg2). The models were subsequently cultivated for 23 days (primary keratinocytes) or 14 days (N/TERT‐2G cells), with the medium changed every 2–3 days.

### 2.5 Fixation of Skin and ESE Models

Skin samples and ESE models were fixed overnight at 4°C in 4% formaldehyde (Thermo Scientific, Massachusetts, USA). Samples were then gradually dehydrated using an ethanol series (50%, 70%, 90%, 95%, 99%, and ROTIHistol (Carl Roth, Karlsruhe, Germany)) and embedded in paraffin (ROTIPlast, Carl Roth, Karlsruhe, Germany). Sections of 8 μm thickness were cut and mounted onto microscope slides. For histological staining, sections were deparaffinized using ROTIHistol (Carl Roth, Karlsruhe, Germany) and rehydrated through a descending ethanol series (99%, 90%, 70%).

### 2.6 Immunofluorescence Staining

For the immunofluorescence staining, skin or ESE sections of 8 μm thickness embedded in paraffin (Carl Roth, Karlsruhe, Germany) were used. Slides were deparaffinized by immersion in ROTIHistol(Carl Roth, Karlsruhe, Germany) for 5 min, followed by rehydration through a descending ethanol series (99%, 90%, 70%), each for 5 min. After washing with PBS, sections were incubated for 20 min with Autofluorescence Reducing Kit Reagent A (Max Block Kit, Dianova, Eching, Germany), then rinsed with 70% EtOH and dipped in 30% EtOH. Antigen retrieval was performed in a pressure cooker using citrate buffer (pH 6.0). After rinsing with PBS, slides were blocked for 45 min with 5% BSA (Santa Cruz Biotechnology, Texas, USA) in PBS. Subsequently, the sections were incubated overnight at 4°C with the primary antibody (see Table 2), followed by washing with PBS. The secondary antibody (see Table 2) was then applied and incubated for 45 min. Slides were washed with PBS and treated for 5 min with Reagent B from the Autofluorescence Reducing Kit (Max Block Kit, Dianova, Eching, Germany). Finally, slides were washed with water, mounted using Roti‐Mount FluorCare DAPI (Carl Roth, Karlsruhe, Germany), and stored at 4°C in the dark until imaging.

**TABLE 2:** Primary and secondary antibodies used in immunofluorescence staining.

Antibodies	Dilution in PBS + 5% BSA	Manufacturer	Product‐ID	Epitope region
Anti‐ALOX12B mouse	1:200	Sigma‐Aldrich^a^	HPA024002‐100UL	C‐terminal
Anti‐CYP4F22 rabbit	1:200	Invitrogen^b^	PA5‐103399	C‐terminal
Anti‐CERS3 rabbit	1:200	Antibodies.com^c^	A43437	N‐terminal
Anti‐Filaggrin rabbit	1:200	Abcam^d^	ab218863 / SPM181	Not disclosed by manufacturer
Anti‐Involucrin mouse	1:200	Sigma‐Aldrich^a^	SAB4200794	Not disclosed by manufacturer
Anti‐GlcCer rabbit	1:200	Glycobiotech^e^	RAS_0011	Not disclosed by manufacturer
Rabbit IgG Alexa Fluor 594	1:500	Invitrogen^b^	A11012	N/A
Mouse IgG Alexa Fluor 594	1:500	Invitrogen^b^	A21203	N/A

^a^ Sigma‐Aldrich, St. Louise, USA.

^b^ Invitrogen, Waltham, USA.

^c^ Antibodies.com Europe AB, Stockholm, Sweden.

^d^ Abcam, Cambridge, United Kingdom.

^e^ Glycobiotech GmbH, Kükels, Germany.

### 2.7 2D Differentiation

Keratinocytes were cultured in Petri dishes (10 cm diameter) until they reached 70%–80% confluence. To initiate the differentiation, E1 medium was supplemented with 1.21 mM CaCl_2_, 0.3 mM palmitic acid (Sigma‐Aldrich, St. Louis, USA) and 15 μM linoleic acid (Sigma‐Aldrich, St. Louis, USA) and then applied to the cells. The medium was changed three times per week. On day 0 (start of differentiation), day 3, day 7, and day 14, RNA was isolated for qPCR analysis.

### 2.8 RNA Analysis

RNA was isolated from keratinocytes during the 2D differentiation using the RNeasy Mini Kit, QIAshredder and on‐column DNase digestion (Qiagen, Hilden, Germany) according to the manufacturer's instructions. Subsequently, cDNA was synthesized using the QuantiTect Reverse Transcription Kit (Qiagen, Hilden, Germany) and PCR was performed according to standard protocols. qPCR was performed in triplicates using SYBR Green reagent (Qiagen, Hilden, Germany). Primer pairs for the amplification of *filaggrin, involucrin, ALOX12B*, *CYP4F22*, *CERS3*, and *ACTB* (housekeeping gene) were obtained from Qiagen (QuantiTect Primer Assay). Primer binding sites are not affected by the mutations. Amplification and fluorescence detection were performed on a LightCycler 480 (Roche, Basel, Switzerland) over 45 cycles. Relative mRNA expression levels were calculated using the 2^−ΔΔCt^ method.

### 2.9 Statistical Analysis

qPCR data were analysed using GraphPad Prism software (version 10.0.2). For statistical analysis, the nonparametric Mann–Whitney test was used.

## 3 Results

### 3.1 Histopathological Analysis of Skin and Skin Models

**FIGURE 1:**
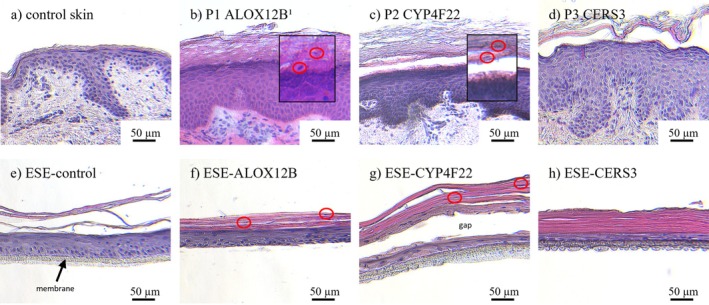
H&E staining of human skin and epidermal skin equivalents (ESEs). Healthy skin as a control was compared to skin from AR‐nEDD patients carrying pathogenic variants in ALOX12B, CYP4F22 or CERS3 (a–d). ESEs were generated using primary keratinocytes from these individuals (e–h). Red circles highlight areas of parakeratosis. (*n* ≥ 3, some stainings have more replicates).

Epidermal skin equivalent (ESE) models were generated using primary keratinocytes from a healthy donor (control‐ESE) and patients carrying variants (see Table 1) in *ALOX12B, CYP4F22*, or *CERS3* (ALOX12B‐deficient‐ESE, CYP4F22‐deficient‐ESE, and CERS3‐deficient‐ESE). H&E staining was performed to assess the structural integrity of the skin models and to compare them with the patients' native skin. Histological analysis of the patients' native skin revealed hyperkeratosis as a common feature, consistent with described observations in AR‐nEDDs [3, 7] (see Figure 1b–d). The stratum corneum thickness was approximately 60.85 μm in P1‐ALOX12B and 78.57 μm in P2‐CYP4F22, both markedly higher compared to the control skin, which was about 10.41 μm thick. Although the stratum corneum of patient P3‐CERS3 measured approximately 23.51 μm and was less pronounced than that of patients P1 and P2, it was still more than twice as thick as in control skin (Figure 1; compare a with d). Additionally, parakeratosis was observed in the skin of P1‐ALOX12B and P2‐CYP4F22 (see red circles in Figure 1b,c), and all patients exhibited acanthosis with a thickened stratum spinosum.

Most histopathological features seen in patients' skin were recapitulated in the ESEs generated from patient‐derived primary keratinocytes. Hyperkeratosis, evidenced by increased stratum corneum thickness (~29.47 μm to ~49.70 μm), was consistently observed in all three ESE models derived from patient cells (ALOX12B‐deficient‐ESE, CYP4F22‐deficient‐ESE, and CERS3‐deficient‐ESE) compared to the control‐ESE (~10.69 μm; see Figure 1e–h). A notable finding was the parakeratosis in both ALOX12B‐deficient‐ESE and CYP4F22‐deficient‐ESE (as highlighted by the red circles in Figure 1f,g). Strikingly, in CERS3‐deficient‐ESE, the layers from the stratum basale to the stratum spinosum appeared poorly developed.

In addition to the models generated with patient cells, we also generated ESEs using N/TERT‐2G keratinocytes (N/TERT‐2G‐ESEs). As already mentioned in the introduction, immortalized cell lines can be used for future applications such as gene editing. We further aimed to assess the suitability of these cells as a control for healthy keratinocytes because immortalized cells are known to be more stable and not passage‐dependent in cell culture. The results of N/TERT‐2G‐ESE are shown in the supplement in Figure S1.

### 3.2 Filaggrin, Involucrin and Glucosylceramide Expression in the Skin and in the ESE

**FIGURE 2:**
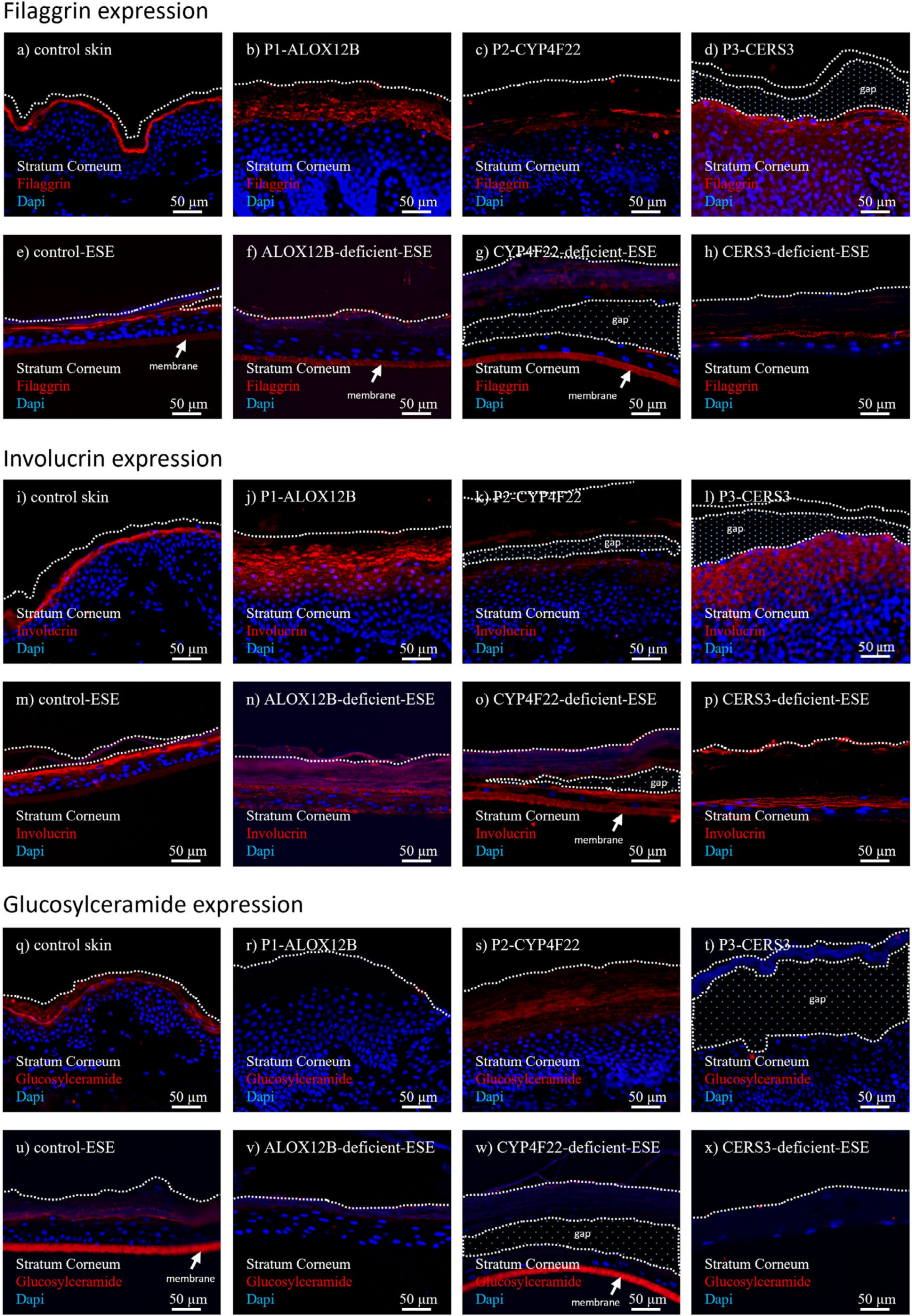
Immunofluorescence analysis of filaggrin, involucrin, and glucosylceramide expression in skin samples and epidermal skin equivalents (ESEs). The dashed line indicates the edge of the stratum corneum or a separation gap. Filaggrin, involucrin, and glucosylceramide were expressed in the stratum granulosum of control skin but were reduced or absent in patient skin. Most skin models mirrored the expression patterns observed in the respective patient samples (Skin models were cultured on polycarbonate membranes; in some cases, the membranes detached during embedding or staining). Patient biopsies were stained three times on independent sections (technical replicates). Three independent ESEs were generated per condition and stained once each (biological replicates).

In healthy skin, profilaggrin—a 350 kDa precursor of filaggrin—is proteolytically processed into 37 kDa filaggrin monomers at the transition of the stratum granulosum to the stratum corneum, as evidenced by filaggrin staining in healthy control skin (see Figure 2a). The filaggrin monomers seem to be degraded into free amino acids in the upper part of the stratum corneum. In P1‐ALOX12B, however, filaggrin expression was observed throughout the lower part of the thickened stratum corneum (see Figure 2b). In P3‐CERS3, filaggrin was even expressed from stratum spinosum to stratum corneum (Figure 2d) while P2‐CYP4F22 showed low levels of filaggrin expression in both the stratum granulosum and stratum corneum (see Figure 2c). Filaggrin expression in the corresponding epidermal skin equivalents (ESEs) generally reflected the observations of the native skin (see Figure 2e–h). In CERS3‐deficient ESE, H&E staining already revealed that the stratum corneum was the most pronounced layer, while the remaining epidermal layers were poorly distinguishable. Consequently, filaggrin expression was reduced compared to native skin, and only a few nuclei were stained (see Figure 2h).

Involucrin is synthesized in the stratum spinosum, where it initially appears in the cytoplasm and is later cross‐linked to membrane proteins, forming part of the insoluble envelope beneath the plasma membrane in the stratum granulosum. Similar to filaggrin, involucrin was localized in the stratum granulosum of healthy skin as a distinctly stained, thin, well‐defined band (see Figure 2i). In comparison, patient P2‐CYP4F22 showed severely reduced involucrin expression (see Figure 2k). In patients P1‐ALOX12B and P3‐CERS3, in contrast, involucrin expression was significantly increased and detectable from the stratum spinosum to the stratum granulosum, showing a broader staining pattern and lacking the sharp boundary seen in the control (see Figure 2j,l). Involucrin expression in control‐ESE (see Figure 2m) closely resembled that of native healthy skin. Similarly, filaggrin expression in ALOX12B‐deficient‐ESE and CYP4F22‐deficient‐ESE matched the corresponding native skin samples, P1‐ALOX12B and P2‐CYP4F22, respectively (compare Figure 2j with 2n, and Figure 2k with 2o). As the epidermal layers in the CERS3‐deficient‐ESE were poorly developed, a thinner band of involucrin was observed beneath the stratum corneum (see Figure 2p).

Glucosylceramide is a glycosphingolipid primarily located in the outermost layers of the epidermis, where it plays a crucial role in the formation and maintenance of the skin lipid matrix. In the control skin, glucosylceramide was most prominently detected in the stratum granulosum, with weak staining extending into the stratum corneum (Figure 2q). In comparison, patient P2‐CYP4F22 exhibited reduced glucosylceramide staining (see Figure 2s), while no staining was observed in patients P1‐ALOX12B and P3‐CERS3 (see Figure 2r,t). In control‐ESE, glucosylceramide staining intensity was significantly lower compared to native skin and confined to the stratum granulosum (see Figure 2u). No glucosylceramide was detected in the skin models generated from patient keratinocytes (ALOX12B‐deficient‐ESE, CYP4F22‐deficient‐ESE and CERS3‐deficient‐ESE) (see Figure 2v–x).

### 3.3 AR‐nEDD Protein Expression in Skin and Epidermal Skin Equivalents

**FIGURE 3:**
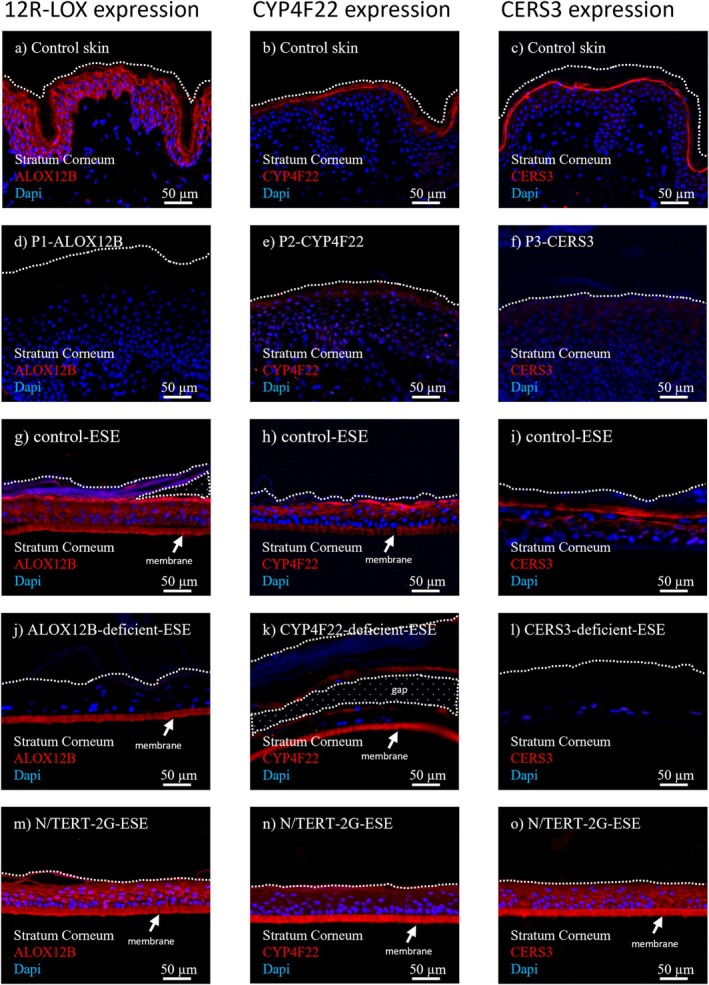
Immunofluorescence staining of ALOX12B, CYP4F22, and CERS3 expression in (a–c) control skin, (d–f) patient skin samples, and (g–o) epidermal skin equivalents (ESEs). The dashed line indicates the edge of the stratum corneum or a gap (Skin models were cultured on polycarbonate membranes; in some cases, membranes detached during embedding or staining). Patient biopsies were stained three times on independent sections (technical replicates). Three independent ESEs were generated per condition and stained once each (biological replicates).

To assess the suitability of artificial skin models generated from patient keratinocytes carrying pathogenic variants in *ALOX12B*, *CYP4F22*, and *CERS3*, we performed immunofluorescence staining on paraffin sections to determine whether these models could reproduce the expression patterns of these three related proteins in the skin. All three proteins selected for this study are part of the same metabolic pathway, which is crucial for the synthesis of barrier lipids. Our staining results in healthy skin confirmed that 12R‐LOX is expressed from the stratum basale to the stratum granulosum [22], while CYP4F22 and CERS3 are primarily expressed in the stratum granulosum of the epidermis (see Figure 3a–c). The control‐ESE reflected the expression patterns of ALOX12B, CYP4F22 and CERS3.12R‐LOX was expressed throughout the entire control‐ESE (see Figure 3g), whereas CYP4F22 and CERS3 were strongly expressed in the stratum granulosum (see Figure 3h,i).

In patients P1‐ALOX12B and P3‐CERS3, the respective proteins were undetectable in the skin (see Figure 3d,f). Almost no CYP4F22 fluorescence was observed in patient P2‐CYP4F22 (see Figure 3e). The pathogenic variants in these patients impaired protein synthesis in the skin, leading to reduced or absent expression. These findings are consistent with previous publications [18, 20, 21]. Similarly, the ESEs showed protein expression patterns that mirrored those observed in the patients' skin samples (see Figure 3j–l). Notably, the number of cell nuclei in the ESEs was markedly reduced compared to the corresponding native skin samples. This may be due to extensive keratinization of keratinocytes, along with a limitation of the 3D model, which includes only the epidermal layer.

Remarkably, the three AR‐nEDD proteins examined here were expressed in ESEs generated with N/TERT‐2G keratinocytes throughout the entire epidermis (see Figure 3m–o). Stronger fluorescence signals of 12R‐LOX and CERS3 were observed in the stratum granulosum. This suggests that N/TERT‐2G keratinocytes express these proteins earlier during epidermal differentiation than primary keratinocytes.

### 3.4 Analysis of the Expression of the Three Studied AR‐nEDD Genes in 2D Cultures of Native and N/TERT‐2G Keratinocytes

**FIGURE 4:**
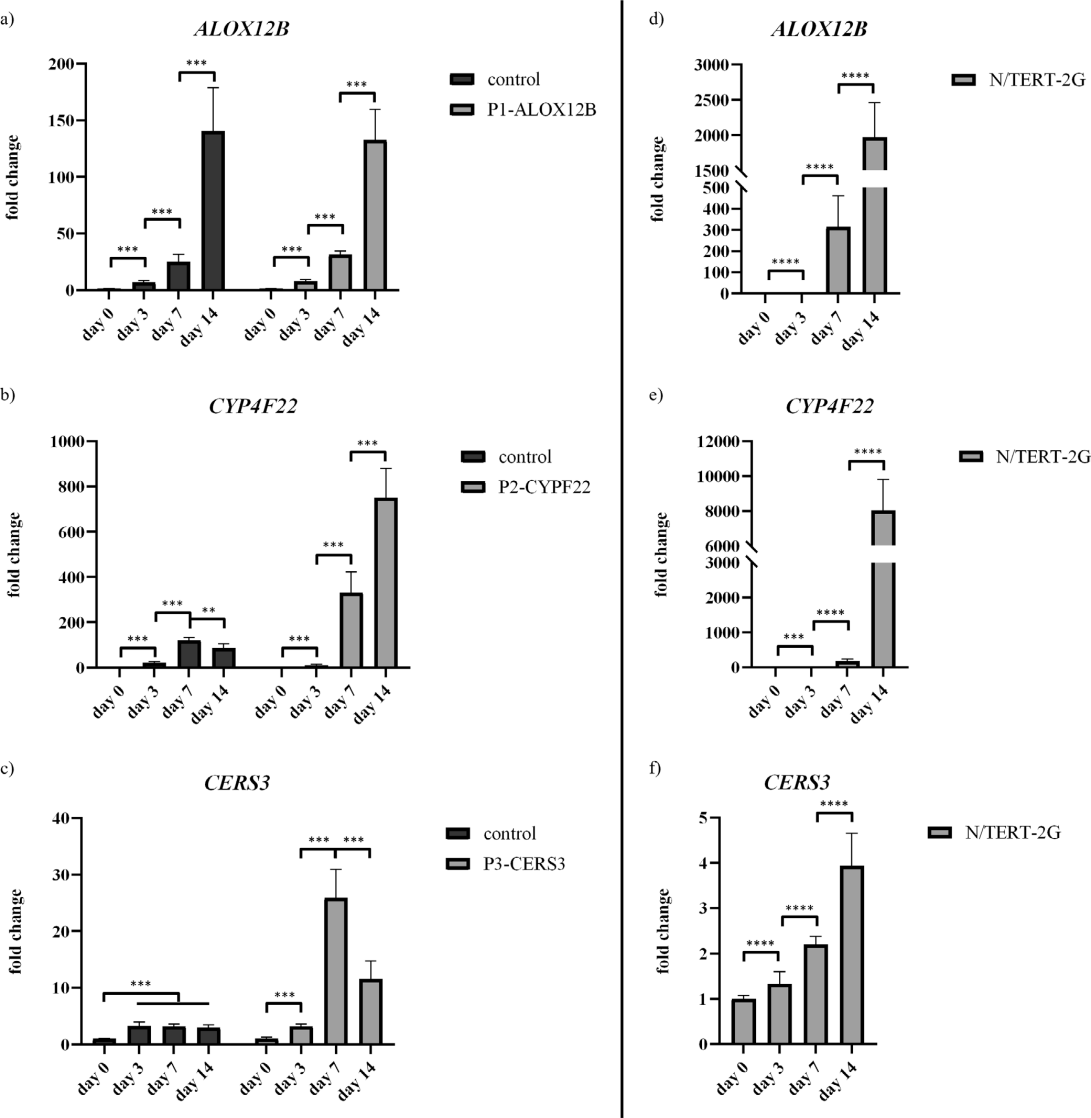
mRNA expression of ALOX12B, CYP4F22, and CERS3 in keratinocytes from a healthy donor and patients (a–c), as well as N/TERT‐2G cells (d–f) during 2D differentiation (the bars represent the standard deviation; **p < 0.01; ***p < 0.001; ****p < 0.0001). Data are presented as mean ± SD from two independent biological experiments (*n* = 2), each performed with technical triplicates (total *n* = 6 measurements per group).

To assess changes in AR‐nEDD gene expression during 2D keratinocyte differentiation in healthy controls and in patients carrying variants in *ALOX12B*, *CYP4F22*, or *CERS3*, we complemented immunofluorescence staining with an analysis of relative mRNA expression. We analysed the relative mRNA expression of AR‐nEDD genes in keratinocytes during 2D differentiation. In both healthy individuals and P1‐ALOX12B, the relative mRNA expression of *ALOX12B* significantly increased during 2D differentiation, showing a four‐ to fivefold increase on day 14 compared to day 7 (see Figure 4a). For P2‐CYP4F22 and P3‐CERS3, the respective mRNA levels on days 7 and 14 were significantly higher than in the healthy control (see Figure 4b,c). In healthy primary keratinocytes, *CYP4F22‐*mRNA expression increased until day 7 and then slightly decreased by day 14. In the control samples, *CERS3‐*mRNA expression rose on day 3 and stayed relatively constant throughout the remaining differentiation period. In patients P2‐CYP4F22, mRNA expression of *CYP4F22* increased continuously until day 14. Moreover, in patients P3‐CERS3, *CERS3*‐mRNA expression was highly elevated on day 7 and then decreases sharply by day 14.

In addition, to gain an overview of AR‐nEDD gene expression in N/TERT‐2G cells, we performed 2D differentiation and analysed mRNA expression. The relative mRNA expression of *ALOX12B* and *CYP4F22* was significantly greater in N/TERT‐2G than in healthy keratinocytes (compare scale of Figure 4a,b with Figure 4d,e). Additionally, the mRNA expression of *CERS3* was increased in N/TERT‐2G, but not as markedly as in the other genes examined (see Figure 4c). Overall, the relative mRNA expression of all investigated genes increased over time during differentiation.

## 4 Discussion

The impact of three‐dimensional skin models has become increasingly important for studying various dermatological disorders. Disease‐specific models are being developed to better understand pathomechanisms and evaluate potential therapeutic strategies. For instance, models for atopic dermatitis and harlequin ichthyosis have been successfully generated using cytokine stimulation, immune cells, or gene‐editing approaches such as CRISPR/Cas9 [16, 22, 23, 24]. Building on previous work with ichthyosis patient‐derived primary keratinocytes [13, 25] this study evaluated the feasibility of generating AR‐nEDD‐specific skin models using both primary keratinocytes harbouring pathogenic variants in ALOX12B, CYP4F22, and CERS3, and the immortalized N/TERT‐2G cell line. It should be noted that the patient‐derived data presented here are based on individual cases, which is an inherent limitation in research on rare genetic skin disorders due to the limited availability of patient material. Rather than drawing broad comparative conclusions, this study is intended as a proof‐of‐concept investigation, demonstrating the potential of epidermal skin equivalents and the N/TERT‐2G cell line as a platform for future research on AR‐nEDDs.

Our histological analyses confirmed that patient‐derived ESEs reproduced key histopathological features of AR‐nEDDs, including hyperkeratosis and parakeratosis, consistent with previous reports [18, 19]. However, acanthosis—present in all patients' native skin—was not recapitulated in the ESEs, likely reflecting the absence of a dermal compartment and immune cell interactions in the epidermal‐only model.

Immunofluorescence staining revealed altered expression patterns of filaggrin and involucrin—two key late differentiation markers essential for skin barrier integrity [26, 27]—in both patient skin and corresponding ESEs, mirroring disease‐specific phenotypes. In patients with pathogenic variants in ALOX12B and CERS3, both filaggrin and involucrin showed early expression extending into the stratum spinosum, a pattern that has also been reported in the context of regenerative immune responses, such as in psoriatic skin [28], and may reflect alterations in protein processing during differentiation [28, 29]. In contrast, filaggrin and involucrin expression was strongly reduced in the patient with CYP4F22 pathogenic variants. CYP4F22 is involved in acylceramide synthesis [20, 30, 31], and it has been suggested that aberrant acylceramide metabolism may secondarily influence the expression of filaggrin and involucrin. The reduced or absent expression of 12R‐LOX, CYP4F22, and CERS3 proteins in patient‐derived ESEs confirmed that these models faithfully replicate the protein expression defects observed in native skin, consistent with previous publications [18, 19, 23, 29]. Notably, glucosylceramide staining was absent in all patient‐derived ESEs, reflecting the disruption of ceramide metabolism caused by the respective pathogenic variants.

Interestingly, despite reduced or absent protein expression, relative mRNA levels of the respective genes were increased in patient keratinocytes during 2D differentiation. This discrepancy may reflect altered transcriptional regulation, compensatory cellular mechanisms, or differences in mRNA stability. Additionally, it should be noted that mRNA expression was assessed in 2D differentiation cultures, whereas protein expression was evaluated in 3D ESEs—two systems that differ considerably in differentiation conditions, cell–cell interactions, and barrier formation—which may further contribute to the observed discrepancy. The precise molecular basis of this observation remains unclear and requires further investigation.

Regarding the N/TERT‐2G cell line, our findings demonstrate that ESEs derived from these immortalized keratinocytes are histologically robust and differentiate faster than primary keratinocytes, requiring only 14 days compared to 23 days. Importantly, NGS panel analysis revealed no known pathogenic AR‐nEDD variants in N/TERT‐2G cells, supporting their suitability as a reliable control system [17]. However, the expression patterns of the three AR‐nEDD‐associated proteins in N/TERT‐2G ESEs differed from those observed in primary keratinocyte ESEs, with earlier expression across epidermal layers, suggesting accelerated differentiation in this cell line. Immortalized keratinocytes are especially advantageous for CRISPR/Cas9 applications, as they are more durable and easier to manipulate than senescent primary cells [16].

In conclusion, both patient‐derived and N/TERT‐2G‐based epidermal models represent valuable and complementary tools for studying AR‐nEDDs. While patient‐derived models remain indispensable for validating disease‐specific phenotypes, N/TERT‐2G keratinocytes offer a reproducible and genetically tractable platform for future gene‐editing approaches [16, 17]. Building upon these findings, we are currently establishing variant‐specific models using genome‐editing techniques, which will provide a standardized platform for mechanistic studies and evaluation of candidate therapeutic strategies.

## Author Contributions

Conceptualization: J.F., H.‐H.H.‐R., C.G.; Data Curation: H.‐H.H.‐R., C.G., C.A., G.W., M.S.; Funding Acquisition: J.F.; Investigation: H.‐H.H.‐R., C.G., C.A., G.W., M.S.; Methodology: H.‐H.H.‐R., C.G., C.A., E.D.; Project Administration: J.F., H.‐H.H.‐R., C.G.; Resource: J.F., N.J., B.K., S.U.E.; Software: C.G.; Supervision: J.F., C.G.; Validation: J.F., H.‐H.H.‐R., C.G., C.A., E.D., G.W., M.S.; Visualization: H.‐H.H.‐R., C.G.; Writing Original Draft Preparation: H.‐H.H.‐R., C.G.; Writing Review and Editing: J.F., C.A., E.D., N.J., B.K., S.U.E.

## Conflicts of Interest

The authors declare no conflicts of interest.

## Supporting information


**Figure S1:** H&E staining of epidermal skin equivalents generated using N/TERT‐2G keratinocytes (N/TERT‐2G‐ESE). The development of N/TERT‐2G‐ESEs was observed on day 7 (a), day 14 (b) and day 21 (c). On day 14, all epidermal layers were most clearly distinguishable.

## Data Availability

All relevant data generated or analysed during this study are included in this published article. The complete datasets used and/or analysed during the current study are available from the corresponding author upon request.
