# Supplementary material for: Potential of 3D Skin Models and N/TERT‐2G Cell Line in Genetic Research on Autosomal Recessive Nonsyndromic Epidermal Differentiation Disorders
Source: Exp Dermatol. 2026 Jun 15;35(6):e70298. doi: 10.1111/exd.70298 (PMC13267678; doi:10.1111/exd.70298)
Supplement: Supplementary file 1 — Figure S1: H&E staining of epidermal skin equivalents generated using N/TERT‐2G keratinocytes (N/TERT‐2G‐ESE). The development of N/TERT‐2G‐ESEs was observed on day 7 (a), day 14 (b) and day 21 (c). On day 14, all epidermal layers were most clearly distinguishable. [file EXD-35-e70298-s001.docx]

**SUPPLEMENTARY MATERIAL**

The development of N/TERT-2G-ESEs was monitored on days 7, 14, and 21 (see Figure S1). Differentiation appeared to occur more rapidly in N/TERT-2G keratinocytes compared to primary keratinocytes. Typically, the standard protocol requires 21 days to produce an ESE. By day 7, the immortalized keratinocytes had already formed multiple layers. Towards the surface (from stratum basale to stratum corneum) the nuclei flattened and began to disappear. By day 14, a well-developed stratum corneum was visible at the surface of the model (see red arrow in Figure S1b). Cells with cuboidal nuclei forming the stratum basale, are visible at the membrane surface (green arrow, Figure S1b). Above the stratum basale, the nuclei of the cells become rounder and begin to flatten, forming a stratum spinosum consisting of approximately three cell layers. Just beneath the pink-stained stratum corneum, a thin stratum granulosum was observed, with dark granular areas appearing in certain regions. After 21 days of culture, the skin model showed reduced epidermal thickness. Keratinization appeared more pronounced, and the stratum basale was less clearly defined compared to day 14 (compare Figure S1b with S1c).


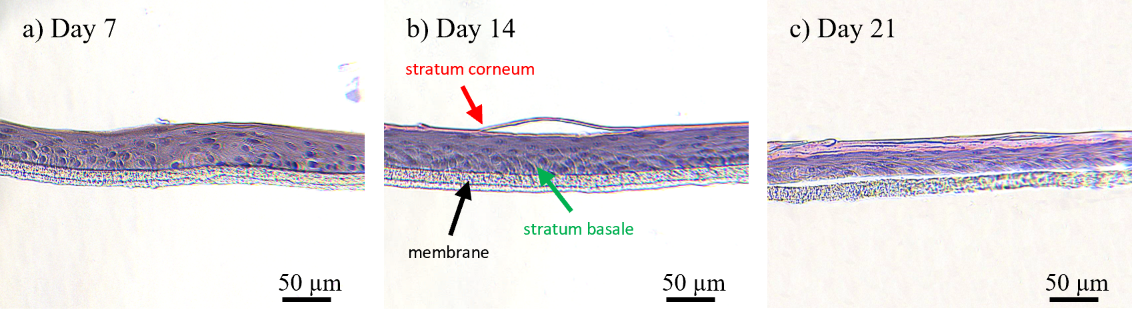


**Figure S1:** H&E staining of epidermal skin equivalents generated using N/TERT-2G keratinocytes (N/TERT-2G-ESE). The development of N/TERT-2G-ESEs was observed on day 7 a), day 14 b) and day 21 c). On day 14, all epidermal layers were most clearly distinguishable.
